# Mr. Paget’s Report Continued

**Published:** 1844-05-04

**Authors:** 


### RECORD OF MEDICAL SCIENCE.

### MR. PAGET'S REPORT CONTINUED.

#### SPECIAL SENSES.

*Eye.*—Dr. W. Clay Wallace has described two new muscles of the eye, to which he has assigned the function of adjusting the focal length of the organ for viewing distant and near objects. They resemble crescents, the horns of which meet at the equator of the eye, and they surround the gray cellular matter connecting the ciliary processes. Their fibres are radiated, and their colour like that of the muscles of the frog's leg. The trunks of the ciliary arteries pass the muscular fibres at the junction of the crescents and are therefore not affected by their contraction; but the veins pass directly under the muscles and are compressed at each act of contraction. Supposing therefore that the eye is adjusted to a remote object and suddenly directed to a near one, an indistinct image of the latter is formed on the retina. The impression of this image is, Dr. C. Wallace thinks, communicated to the sensorium, and by a reflex impression through the third and fifth nerves the muscles around the ciliary processes are made to contract; the veins of those processes being thus compressed they become erect, and their apices which float in the aqueous humour are elongated: these apices being attached to the anterior wall of the canal of Petit, draw it forward and with it the crystalline lens, till a distinct image of the object is formed on the retina. The return of the crystalline to its place is effected by the relaxation of the muscles, the emptying of the veins, and the elastic retraction of the tissue of the vitreous humour.

The description just given of the muscles is from the eye of the ox: in man they form not two crescents, but an entire ring.

According to Dr. Power, the nervous fibres proceeding from the optic ganglia to the retinæ in the loligo, cross each other's course: those from the back part of the ganglia pass on to the anterior part of the retina, and *vice versa*, the bundles interlacing in the most perfect manner, and like the crossing of the fingers of both hands, passing between one another from one side of the ganglion to the opposite side of the retina. Led by this to the examination of the same nerves in higher animals, he found that a similar arrangement existed in all which he examined; in all, either by interlacement or by a half spiral turn, all the filaments which at its origin are in the upper part of the optic nerve, become, near its retinal end, inferior, so that the inferior filaments of the retina correspond to the superior filaments in the brain.

He believes that this arrangement accounts for erect vision, although the impression of an object on the retina must be reversed. And if the observations be true, it is probably not necessary to look further for the explanation of this controverted question.

GENERATION.

*Testicles.*—Mr. Gulliver has confirmed R. Wagner's observation, that the general enlargement of the testicles, which takes place as the period of procreation approaches, is accompanied by enlargement of the individual seminal tubes. During winter he finds that the seminal tubes of birds are tolerably thick and strong; but at the season of procreation semen accumulates in them, and their coats are so distended and attenuated that they are very easily ruptured. The same thinning and enlargement of the tubules occurs in the development of the human testicles at puberty.

An interesting case, proving the sympathy of the vital organs with the testicles, is recorded by Dr. Schlesier. A healthy man engaged in a fray in the dark, was suddenly heard to shriek out: he fell in convulsions and died in five minutes. On examination the only injury found was the rupture of both the spermatic arteries and veins at the internal rings, produced by the scrotum and testicles having been seized and pulled down by one of those with whom the man was fighting.

*Spermatozoa.*—Facts of much importance in regard to the formation of spermatozoa are furnished by the cases first recorded by Mr. Liston and Mr. Lloyd, and since repeatedly observed, in which these bodies are found in the fluid of common hydrocele of the tunica vaginalis testis, and in encysted hydrocele.

*Uterus.*—In an appendix to his former papers on the nervous system of the uterus, Dr. Lee has published a further and very elaborate account of his dissections. The description is not such an one as can be here condensed, but in referring to his original papers, I may be allowed to state, that an examination of the preparations which I have been recently permitted to make, has convinced me that Dr. Lee's account and delineations of them are accurate and complete, and that there can be no reasonable doubt that the structures which he has displayed, are, as he describes them, the nerves and nervous ganglia of the pregnant uterus.

*Ovum—its developement, discharge, &c.*—The Report of Mr. T. Wharton Jones 'On the Ovum of Man and the Mammifera,' inserted in the October number of this Journal, is so complete to the time of its publication, that little need be now said on this department of physiology. I shall only state at greater length the conclusions recently arrived at respecting the escape of ova independently of fecundation, and the connexion of this occurrence with menstruation, which Mr. Wharton Jones was obliged to compress within the limits of a postscript. Many of these conclusions have been long held on insufficient grounds: those which may now be deemed established are as follows:

1. Each act of menstruation is connected with the maturation and discharge of an ovum. Numerous cases in proof of this are related (in addition to those formerly recorded by him and by MM. Gendrin, Negrier, and others,) by Dr. Robert Lee; others by Mr. Girdwood. M. Raciborski has four times found that ova had been recently discharged from the ovaries of virgins who died at or near the period of menstruation; and Bischoff has also four times found Graafian vesicles, containing effused blood, in girls who had recently menstruated.

This menstrual discharge of an ovum is said by Raciborski and Bischoff to be followed by the formation of a corpus luteum, similar to that which is formed when the ovum is impregnated and developed. [But in this I have no doubt they are mistaken. If it were so, one or more corpora lutea should be found in the ovaries of all who die while the habit of menstruation continues; for the *corpus luteum* which forms when impregnation has taken place, is distinct not only through the pregnancy, but for more—often much more—than a month after delivery. Neither are the cavities which are left after the menstrual discharge of ova, or the processes by which they are closed, at all similar to those found when impregnation has taken place. In many examinations of ovaries I have not yet seen a case in which, without impregnation, anything has been found which could be mistaken for a *corpus luteum* formed after an ovum has been discharged and impregnated.] Mr. Girdwood believes that the cicatrices left after the discharge of menstrual ova may be counted, so as to indicate the number of ova discharged and the number of times of menstruation. [But recently I have examined a case in which a girl of seventeen had not menstruated for four months before her death, but previously had menstruated regularly: the ovaries showed no traces of cicatrices. Probably, therefore, the cicatrices remain for a time distinct, but are gradually obliterated, as they are in the nearly analogous case of the discharge of the Peyer's and solitary glands of the intestines.]

3. The menstruation of women, in so far as the periodical maturation and discharge of ova is concerned, is analogous to the *heat* or *rut* of animals. The phenomena, according to Raciborski, may be most distinctly seen in the sow; but in all the domestic mammalia at their period of heat one or more follicles attain their highest degree of development, project upon the surface of the ovary, and at length burst with hemorrhage into their containing cavities, and this whether copulation have taken place or not. Bischoff also has repeatedly found the same things occur in bitches and rabbits whose uterus and tubes have been extirpated; they have heat, the ova mature and detach themselves and pass into the remaining portion of the tube, but of course cannot be impregnated.

4. The discharge of the ova and their passage along the tubes is independent of impregnation and the passage of the seminal corpuscles. This is evident from the facts already mentioned; and others are furnished by Bischoff. In one experiment he kept a bitch carefully secluded till the period of heat ensued. She then copulated once, and immediately after he extirpated the left uterine horn, ovary and oviduct. The copulation had lasted a quarter of an hour; and he found that the semen had penetrated to the upper angle of the uterine horn, but not into the tube. He found also five ova in the oviduct more than two inches from its abdominal orifice; a distance sufficiently great to prove that they had not been detached in the copulation. Next day he killed the bitch, and found that spermatozoa had reached about a quarter of an inch in the right tube; he found also five ova in the same tube.and as many *corpora lutea* in the right ovary, but none of the spermatozoa had come in contact with the ova. These cases prove the detachment of ova before copulation. In some others Bischoff found that they were not detached till long after the act. In some he found that they were undetached twenty-four hours after copulation, and that the seminal corpuscles had passed on towards them. In others also he found the independence of the passages of the ova and the semen still more marked; for example, several days after copulation, ova were found fecundated in one tube. but in the other spermatozoa alone, none of the Graafian vesicles in the corresponding ovary being either enlarged or fully developed.

5. Thus, according to the period of heat at which copulation takes place, will be the place at which the semen meets the ovum. If it be early, the ovum may not escape before the semen reaches the ovary; if late, the ovum may have arrived at the uterus; and probably if it have arrived at the lower or uterine third of the tube before it comes in contact with the semen, impregnation is impossible on account of the changes which the vitellus has already undergone. In women it is in like manner near the period of menstruation that impregnation is most likely to occur. It may take place just before menstruation if the ovum be just mature when the semen reaches the ovary; or some days after, the ovum after its discharge remaining impregnable till the semen reaches it. Or, again, as many analogous circumstances in lower animals prove, an ovum may by the sexual excitement be hurried on to its maturity and discharged; and so, in unusual cases, impregnation may take place at a greater than usual distance from the menstrual period. Still the most common time must be, as common observation shows it is, either during or very near the menstrual period. M. Raciborski has found that in one hundred women there are not more than six or seven in whom this is not the constant rule.

6. All these circumstances prove a closer analogy than was supposed to exist between the discharge of the ova of mammalia and those of the fish, batrachia, and others in which the ova are discharged from the body and impregnated external to it. In all alike the discharge of the ova is an independent act; the differences are in the distances from the ovaries at which the semen is usually brought into contact with it.

Dr. J. E. Panck has published an essay on a case in which he believes that he has made the discovery of the organic connexion between the fallopian tube and the ovary of the human female soon after connexion. A girl, twenty-three years old, was suffocated by carbonic acid five days (it was supposed) after her first conception. There were signs of turgescence about all the uterine and ovarian vessels; the uterus itself was large and vascular, and thickly lined by mucus like a decidua and by ciliary epithelium-cells. On the right side the fallopian tube was turned backwards, and its fringe was expanded over the ovary. They were held together by a fine transparent membrane, which extended over them, over the posterior surface of the uterus and right broad ligament, and a little over the left broad ligament, and was slightly adherent to them all. The left tube and ovary were natural; the right ovary was large and vascular. Directly below the attached fimbriæ there was a cavity in the right ovary, like a distended Graafian vesicle, covered only by serous membrane, about three lines in diameter, and containing a blackish substance like clotted blood. But neither in this nor any where else was an ovum found; so that the evidence of the case is far from complete.

